# Empowering sustainable futures: The role of digital citizenship for health in healthcare and environmental resilience

**DOI:** 10.1371/journal.pdig.0000828

**Published:** 2025-04-18

**Authors:** Sohayla Eldeeb, Salman Fitrat Khan, Lynn-Everdene Bufu Akisarl, Charlotte Anne-Marie Helena Thibault, Maryam Sherif, El Ghali Ouali Alami, Alfredo Lorenzo Recio Sablay, Leonardo Bolstad (Reo Aoki)

**Affiliations:** 1 Digital Transformations for Health Lab (DTH-Lab), Faculty of Medicine, University of Geneva, Genève, Switzerland; 2 Stanford Prevention Research Center, Stanford Medicine, Palo Alto, California, United States of America; 3 Department of Dental Medicine, Faculty of Medicine and Biomedical Sciences of University Yaoundé, Yaoundé, Cameroon; 4 Department of Public Affairs, Sciences Po, Guillaume City, France; 5 Department of Medicine, Faculté de Médecine, Pharmacie et Médecine Dentaire de Fès, Fez, Morocco; 6 Philippine College of Lifestyle Medicine, IloIlo City, Philippines; 7 College of Arts, Science, and Education, St. Paul University Quezon City, New Manila, Philippines; McGill University, CANADA

Digital Citizenship for Health (DC4H) [1] (Table 1) is not merely an emerging concept but an urgent necessity in a world where health systems are becoming increasingly digitalized. It encompasses digital health, and civic literacy, equipping citizens with the agency to engage with digital health systems, advocate for their rights, and co-create ethical governance structures. However, without deliberate investment and citizen-centered approaches, digital transformations risk deepening existing health inequities instead of addressing them. As climate change and environmental degradation accelerate, integrating sustainability into digital health governance is no longer optional—it’s imperative. This commentary explores the key role of DC4H in promoting sustainable healthcare and environmental resilience, calling for bold, multi-stakeholder action and systemic investments in literacy and governance.

**Table 1:** Key conceptual terms mentioned in the opinion piece with definitions and/or explanations.

Term	Definition/Explanation
Digital Citizenship For Health (DC4H)	Digital citizenship for health builds on the concept of digital citizenship while accounting for the profound effects of digitalization on the health and well-being of societies [Source: Digital Transformations for Health Lab (2025) Brief: Digital Citizenship for Health. Geneva; DTH-Lab]
Digital Health	The use of data and communicating technologies, such as telemedicine, electronic health records, and artificial and augmented intelligence, to improve and optimize healthcare delivery.
Climate Change	Article 1 of the United Nations Framework Convention on Climate Change (UNFCCC) defines climate change as “a change of climate which is attributed directly or indirectly to human activity that alters the composition of the global atmosphere and which is in addition to natural climate variability observed over comparable time periods.”
Triple Planetary Crisis	The interconnected global challenges of climate change, pollution, and biodiversity loss, which threaten environmental stability, human health, and the sustainability of ecosystems and societies.
Climate Adaptation	Adjustment to actual or expected climate and its effects.
Climate Mitigation	Efforts to reduce emissions (of greenhouse gasses)
Alliance for Transformative Action on Climate and Health (ATACH)	A global initiative that brings together a network of WHO Member States and other stakeholders to realize the ambition set at COP26 to build sustainable and climate-resilient health systems, ensuring that healthcare policies and infrastructure align with climate adaptation and mitigation goals.
Youth Engagement	Actively and meaningfully involving young people’s ideas, perspectives, and experiences in decision-making processes, policy advocacy, and programs to drive social, environmental, and health-related change, recognizing them as key stakeholders in their own development.

The triple planetary crisis—climate change, pollution, and biodiversity loss—poses an existential threat to global health and is worsening respiratory diseases, vector-borne illnesses, and food and water insecurity [2]. Climate change is already undermining key environmental and social determinants of health and is projected to cause 21 million deaths by 2050 [3]. Healthcare systems contribute to approximately 5% of carbon emissions through energy use, medical waste, and unsustainable supply chain operations [4]. Failure to adopt greener practices deepens the environmental and public health crisis, disproportionately impacting marginalized regions. Integrating sustainability into healthcare is crucial to mitigating climate-related health risks and strengthening system resilience. Digital health innovations, including telemedicine, mobile applications, AI-powered diagnostics, and electronic health records, offer tangible opportunities to optimize resource use and fortify healthcare systems against the challenges posed by the triple planetary crisis [5]. However, their deployment must be consciously aligned with sustainability principles. DC4H plays a transformational role by equipping individuals and communities with the knowledge and tools necessary to demand and drive green healthcare policies.

DC4H extends traditional citizenship concepts into the digital health sphere, centering on individuals’ active and informed participation in digital health governance. It goes beyond digital literacy, thereby framing health engagement as a civic responsibility and enabling individuals to navigate digital health systems, critically analyze technologies, combat misinformation, and influence health policies. By managing their health data and advocating for equitable digital policies, engaged citizens actively participate in addressing health disparities and prioritizing the needs of the most vulnerable.

Citizens can better champion DC4H through system-wide transformations—from advocating for climate-resilient healthcare facilities to promoting renewable energy adoption in medical infrastructure. Despite the escalating risks caused by climate change, healthcare systems worldwide remain unprepared for the impending crisis. Prioritizing both climate adaptation and mitigation strategies saves lives in the short term and also strengthens long-term resilience and reduces inequities. Supporting country-led plans to develop climate-responsive, resilient, and low-carbon health systems and harnessing the collective power of all stakeholders through networks like the Alliance for Transformative Action on Climate and Health (ATACH) is an ethical and strategic necessity for ensuring preparedness against emerging threats [6].

Amongst all stakeholders, youth have a critical role to play in this transformation. As the generation that will inherit the consequences of today’s climate decisions, young people are not passive beneficiaries. They are active stakeholders in shaping sustainable digital health solutions [7]. As digital natives, their involvement in policy dialogs, digital health literacy initiatives, and the co-creation of technological innovations is non-negotiable. Failing to integrate youth perspectives into digital health governance and give them agency in policy processes weakens our ability to build an equitable, climate-resilient future. Instead of relying on unscalable innovations in the name of “youth engagement,” we must prioritize meaningful inclusion [7].

A major roadblock to sustainable healthcare is the phenomenon of “health-washing,” where organizations make superficial or misleading claims about their sustainability efforts while engaging in business-as-usual activities. At major events such as the UNFCCC Conference of Parties (COP) 28, countries have engaged in healthwashing by incorporating health-related language into climate negotiations, making “pro-health” commitments while continuing to support harmful practices, such as the fossil fuel industry, that undermine public health [8]. When corporations exaggerate eco-friendly commitments, citizens may be misled about actual health benefits of certain digital platforms or technologies, affecting informed decisions—an essential DC4H component. Health-washing leads to public disengagement from digital health platforms and can disproportionately affect vulnerable populations who rely on accurate information to access equitable care.

Addressing health-washing requires robust accountability frameworks. Policymakers must enforce transparent reporting mechanisms and legally binding sustainability benchmarks to ensure that environmental commitments are not a marketing gimmick. Addressing health-washing and promoting collaboration among healthcare providers, youth, researchers, and civil society are essential to building public trust. These efforts encourage meaningful engagement in digital health citizenship, helping us push for digital transformations that prioritize both public health and the planet.

## Recommendations and call to action

To fully harness the potential of DC4H in advancing sustainable healthcare and environmental resilience, deliberate, collective action is a must. Key stakeholders must step up:

- Youth must actively participate as leaders and co-creators of climate-resilient health systems by amplifying advocacy efforts, enhancing their digital health literacy, and influencing policy decisions.
- Healthcare professionals must champion digital health technologies that do no harm to the environment and promote adaptive solutions in response to climate threats.
- Policymakers must act decisively by prioritizing digital equity, strengthening regulations to combat data misuse and health-washing, and investing in green health technologies.
- Governments, industry leaders, the private sector, and civil society organizations must collaborate to ensure that digital health systems are sustainable, inclusive, and accessible, especially in low- and middle-income countries and regions with high youth populations.

The window for meaningful action is closing. DC4H is a powerful tool for ensuring sustainable healthcare and environmental resilience amidst the ongoing triple planetary crisis. By advancing digital, health, and civic literacy, DC4H empowers individuals to demand climate-smart solutions, advocate for equitable policies, advance digital health transformations while ensuring environmental sustainability, and participate in ethical digital health governance. However, this transformative potential will remain unrealized unless bold action is taken now. Digital health systems must be designed with planetary well-being and DC4H at its core because the cost of inaction is simply too great to ignore.
